# Supplementary material for: Genetic diagnosis of Mendelian disorders via RNA sequencing
Source: Nat Commun. 2017 Jun 12;8:15824. doi: 10.1038/ncomms15824 (PMC5499207; doi:10.1038/ncomms15824)
Supplement: Supplementary Information — Supplementary Figures, Supplementary Tables, Supplementary Notes, Supplementary Methods and Supplementary References [file ncomms15824-s10.pdf]

Supplementary Information

All figures and tables are also available at our webserver:

https://i12g-gagneurweb.informatik.tu-muenchen.de/public/paper/mitoMultiOmics/

Supplementary figures

1. Supplementary Figure

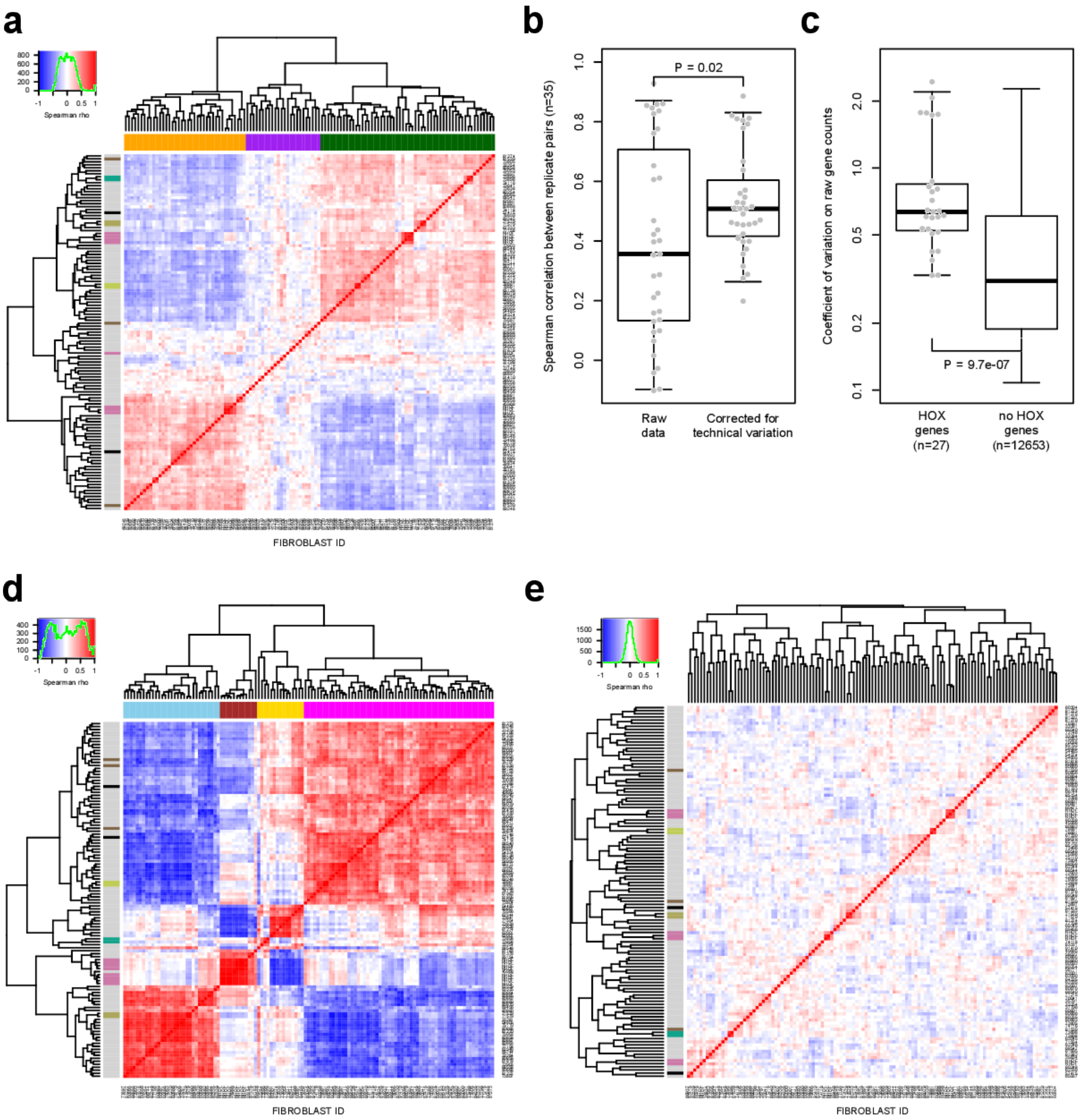

RNA normalization. (a) Spearman correlation heat map of size-factor normalized gene expression between all fibroblasts (n=119) including biological replicates (left side color code). The dendrogram represents the sample-wise hierarchical clustering. The color code on the top depicts the top three clusters. The color key of the spearman rho value (top left) includes a histogram based on the values (green line). (b) Boxplot of the spearman correlation between all replicate pairs (n=35) before and after normalizing for technical variation. Equi-tailed 95% interval (whiskers), 25th, 75th percentile (boxes) and median (bold horizontal line) are indicated. The *P*-value is based on a two-sided Wilcoxon test. (c) Boxplot as in (b) of coefficients of variation (standard deviation / mean) for the 30 HOX genes and the remaining genes based on raw gene counts. The *P*-value is based on a two-sided Wilcoxon test. (d) Same as (a), but correlation is computed only on the HOX genes among all samples. The top four clusters are highlighted (color code top). (e) Same as (a) after normalization for the technical variation, sex variation, and four HOX gene groups.

2. *Supplementary Figure*

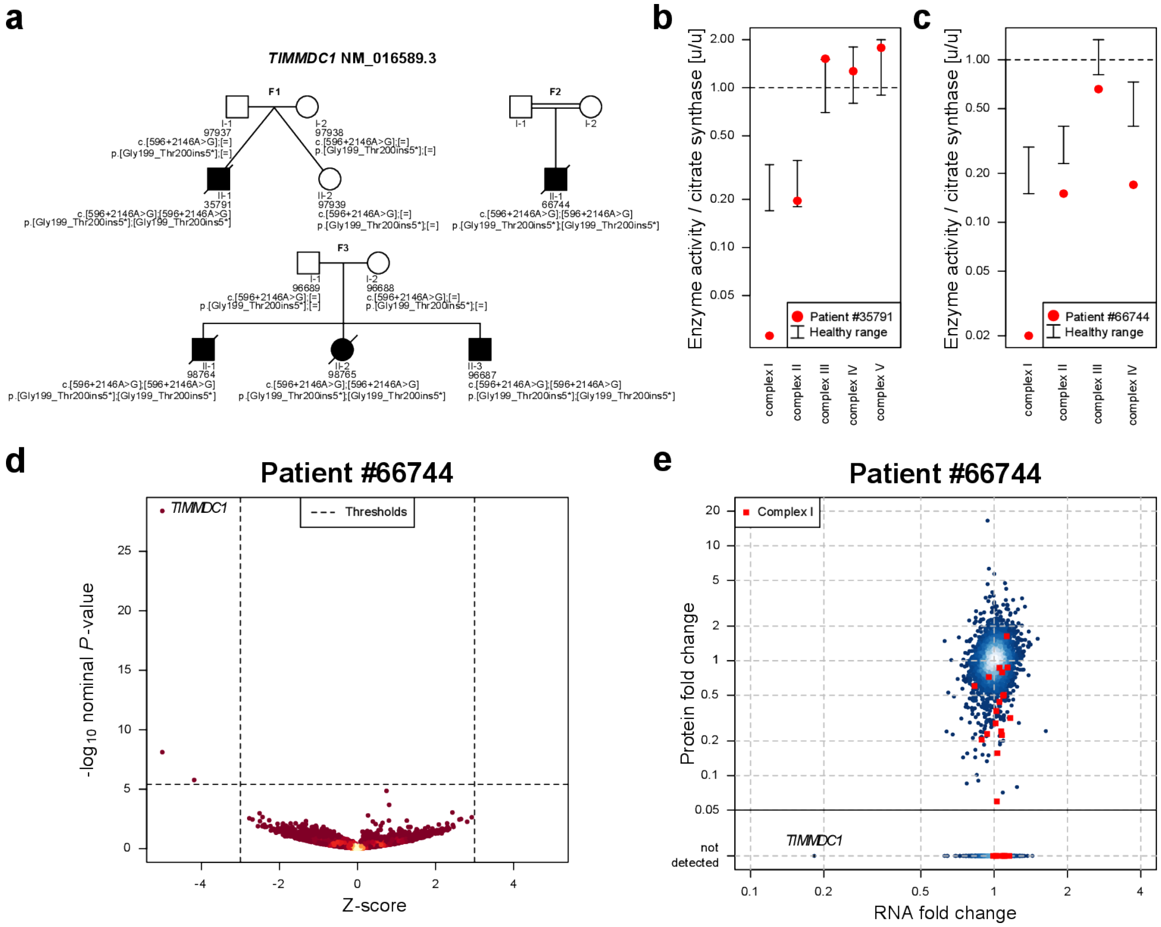

TIMMDC1. (a) Pedigrees of the families with mutations in *TIMMDC1* showing the mutation status. Mutations were confirmed by Sanger sequencing. (b) Enzyme activities of respiratory chain complexes I-V of #35791. Activities were measured in a muscle biopsy and normalized to citrate synthase. (c) Enzyme activities of respiratory chain complexes I-IV of #66744. Analogous to (b). (d) Gene-wise RNA expression volcano plot of nominal *P*-values (- $\log_{10}$ *P*-value) against Z-scores of the patient #66744 compared against all other fibroblasts. Absolute Z-scores greater than 5 are plotted at ±5, respectively. (e) Gene-wise comparison of RNA and protein fold changes of the patient #66744 against all other patient fibroblasts. Reliably detected proteins that were not detected in this sample are shown separately with their corresponding RNA fold changes (points below solid horizontal line). Subunits of the mitochondrial respiratory chain complex I are highlighted (red squares).

3. Supplementary Figure

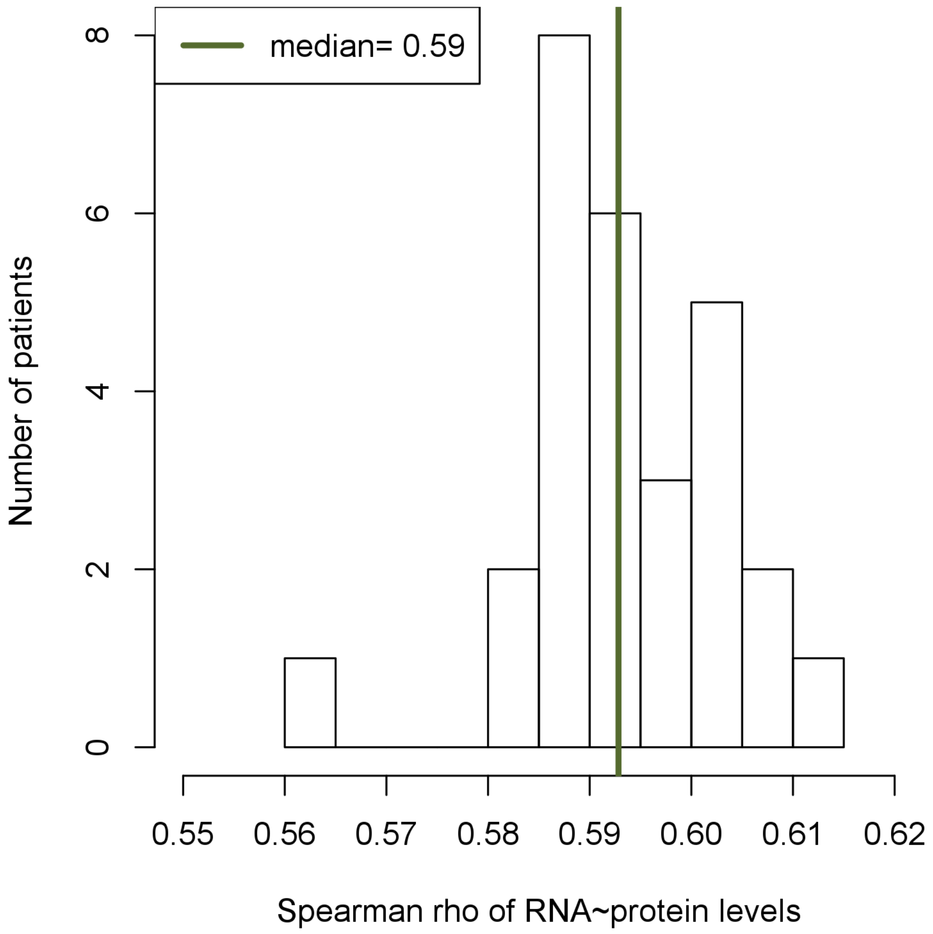

Histogram of spearman correlation of RNA and protein levels for each patient with proteomics data available (n=31). Median is highlighted (green line).

4. Supplementary Figure

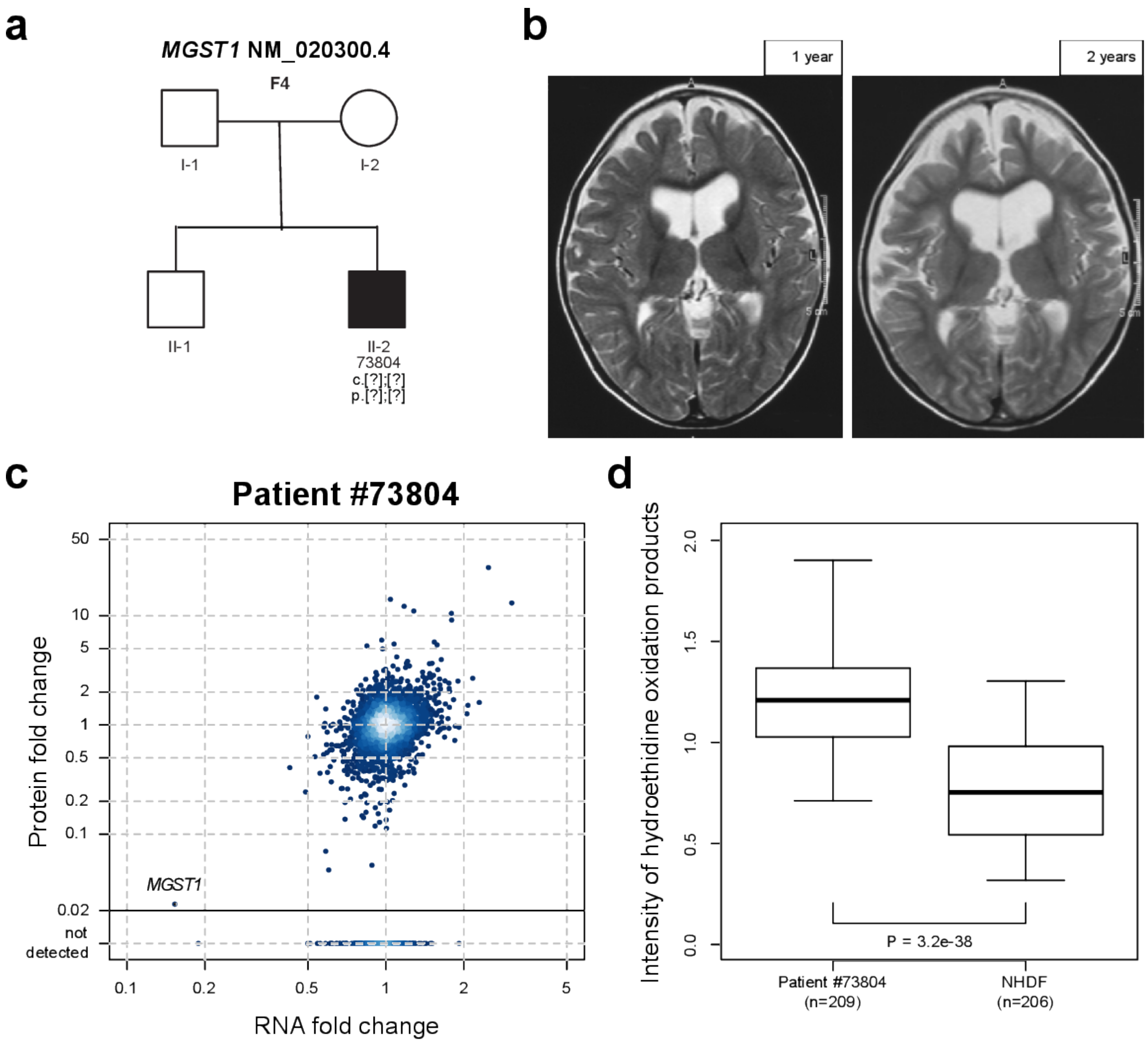

MGST1. (a) Pedigree of the family with mutations in *MGST1* showing the mutation status. (b) Magnetic resonance imaging of the brain of patient #73804 at the age of one and two years (left and right panel, respectively). (c) Gene-wise comparison of RNA and protein fold changes of the patient #73804 against all other patient fibroblasts. Reliably detected proteins that were not detected in this sample are shown separately with their corresponding RNA fold changes (points below solid horizontal line). (d) Quantification of cellular ROS production. Hydroethidine oxidation production was measured using epifluorescence microscopy. Equi-tailed 95% interval (whiskers), 25th, 75th percentile (boxes) and median (bold horizontal line) are indicated. The *P*-value is based on a two-sided Wilcoxon test.

5. Supplementary Figure

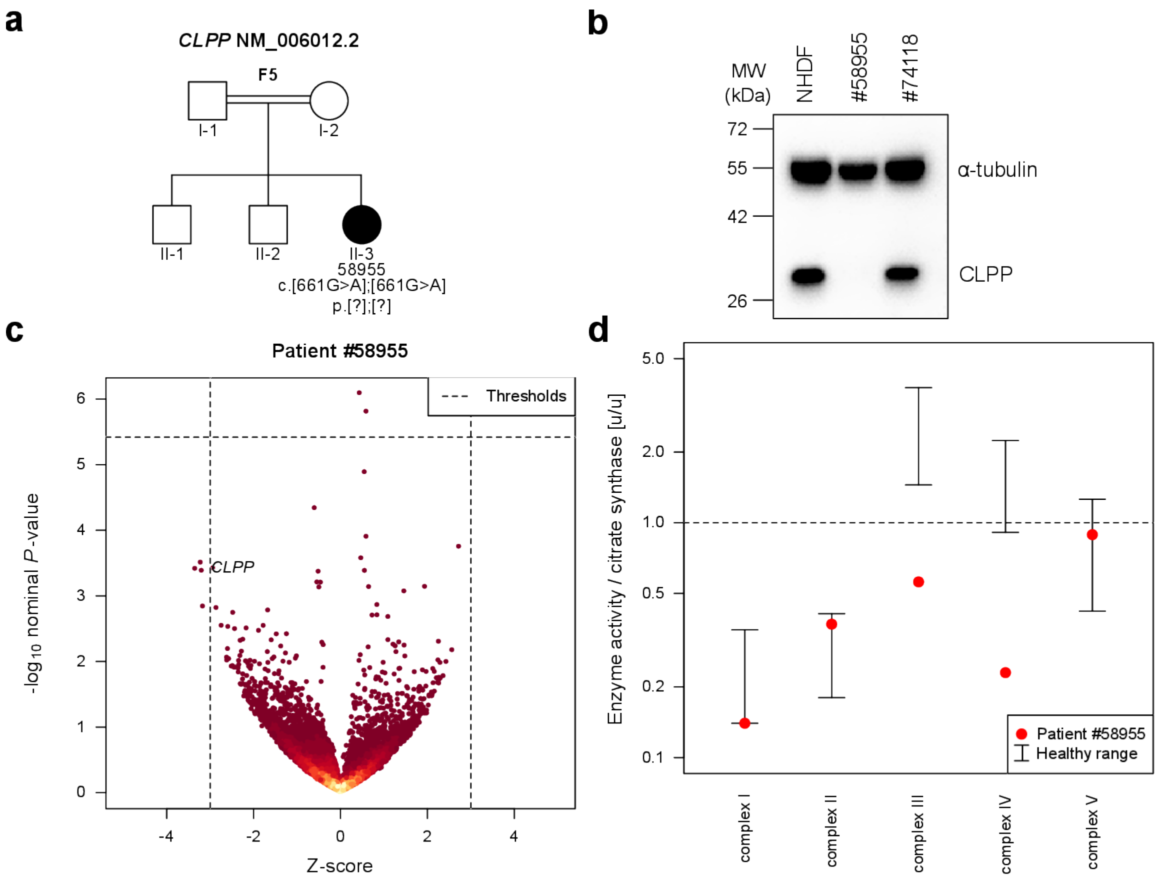

CLPP. (a) Pedigree of the family with mutations in *CLPP* showing the mutation status. (b) Western Blot showing the amount of CLPP for the NHDF cell line, the patient carrying variants in *CLPP* (#58955), and a patient not carrying variants in *CLPP* (#74118). α-tubulin was used as loading control. (c) Gene-wise RNA expression volcano plot of nominal *P*-values (- $\log_{10}$ *P*-value) against Z-scores of the patient #58955 compared against all other fibroblasts. Absolute Z-scores greater than 5 are plotted at ±5, respectively. (d) Enzyme activities of respiratory chain complexes I-V of #58955. Activities were measured in a muscle biopsy and normalized to citrate synthase.

6. *Supplementary Figure*

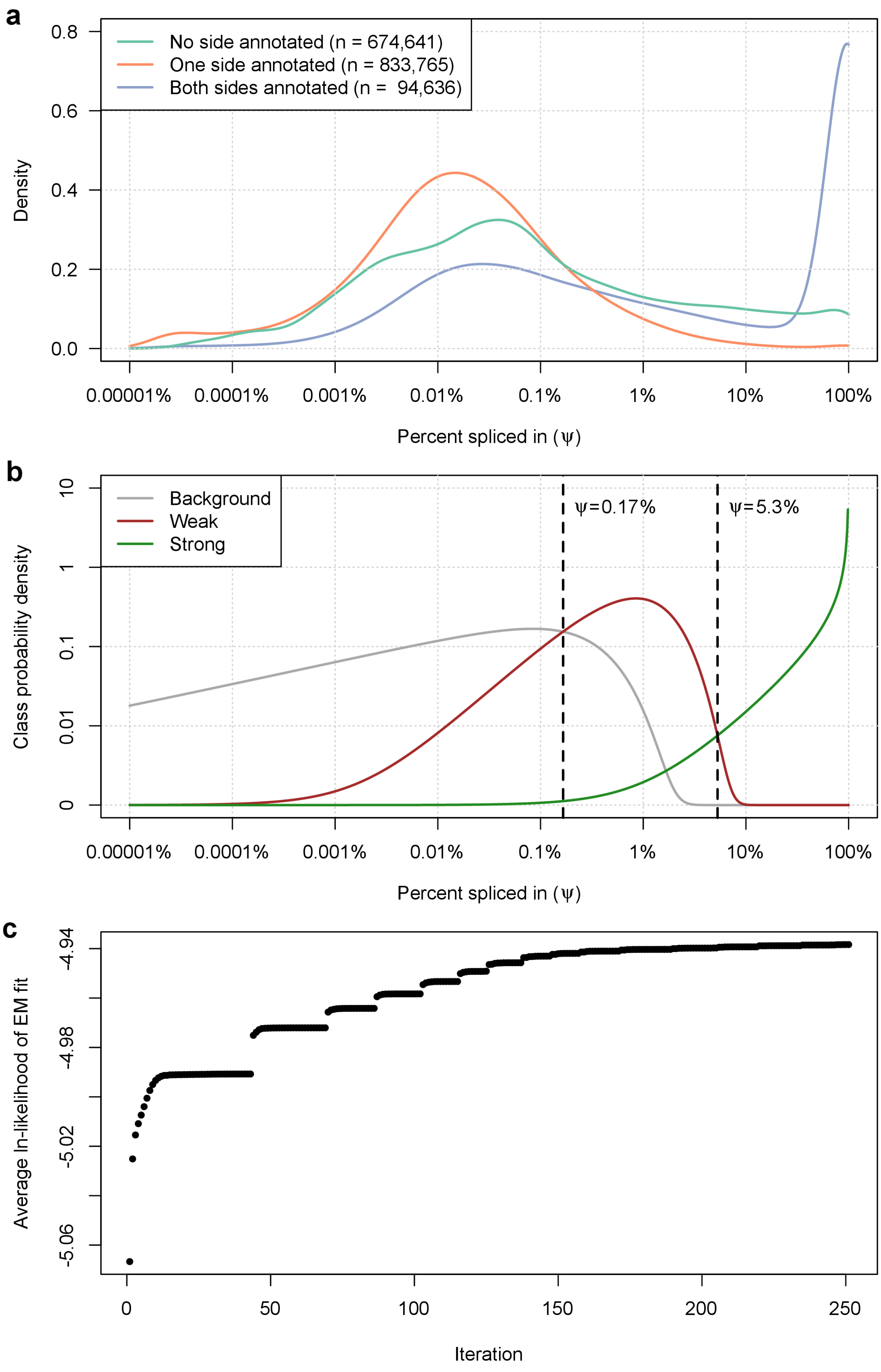

Percent spliced in distributions. (a) The densities of genome-wide percent spliced in (Ψ) 5' and 3' values grouped by their GENCODE annotation status: Both sides of junction are annotated (green), only one side of the junction is annotated (orange), and no side of the junction is annotated (blue). (b) The expectation maximization (EM) fitted splice class model based on the GENCODE annotation status. Each line represents the probability density belonging to a splice class given a Ψ-value. (c) The convergence of the EM algorithm. Each point represents the average ln-likelihood of the EM-fit after a specific iteration cycle (n=250).

7. *Supplementary Figure*

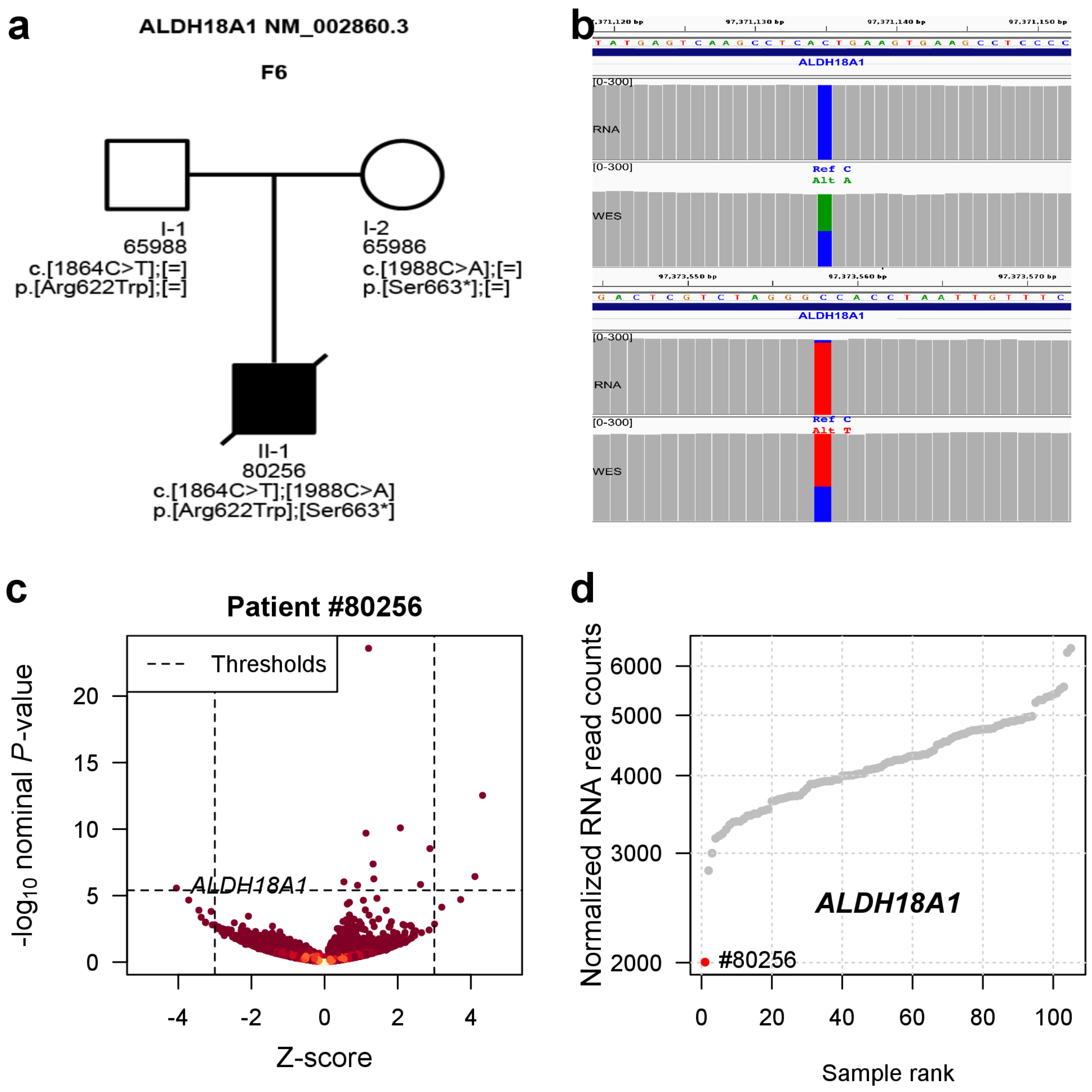

ALDH18A1. (a) Pedigree of the family with mutations in *ALDH18A1* showing the mutation status. Mutations were confirmed by Sanger sequencing. (b) Exome and RNA sequencing read coverage tracks (gray) of the two SNVs indicated in (a) for *ALDH18A1* (antisense strand). Alternative (Alt) and reference (Ref) nucleotides are indicated by their corresponding color (A green, G brown, T red, C blue). (c) Gene-wise RNA expression volcano plot of nominal *P*-values (- $\log_{10}$ *P*-value) against Z-scores of the patient #80256 compared against all other fibroblasts. Absolute Z-scores greater than 5 are plotted at ±5, respectively. (d) Sample-wise RNA expression is ranked for *ALDH18A1*. Samples with aberrant expression for the corresponding gene are highlighted in red (#80256).

8. *Supplementary Figure*

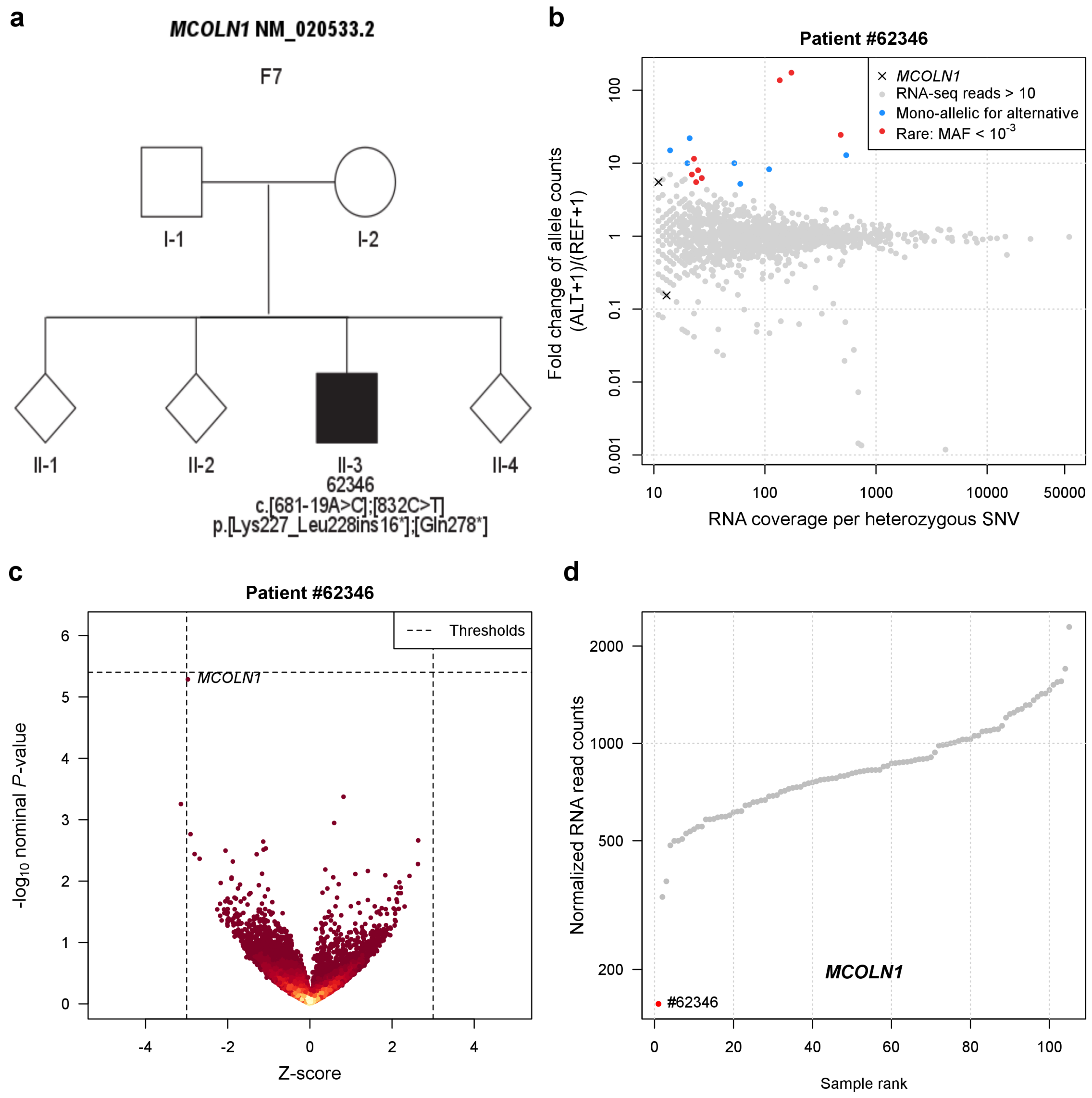

MCOLN1. (a) Pedigree of the family with mutations in *MCOLN1* showing the mutation status. (b) Fold change between alternative (ALT+1) and reference (REF+1) allele read counts for the patient #62346 compared to total read counts per SNV within the sample. Points are colored according to the steps of the mono-allelic expression variant filter cascade. (c) Gene-wise RNA expression volcano plot of nominal *P*-values (-log10 *P*-value) against Z-scores of the patient #62346 compared against all other fibroblasts. Absolute Z-scores greater than 5 are plotted at ±5, respectively. (d) Sample-wise RNA expression is ranked for *MCOLN1*. Samples with aberrant expression for the corresponding gene are highlighted in red (#62346).

9. Supplementary Figure

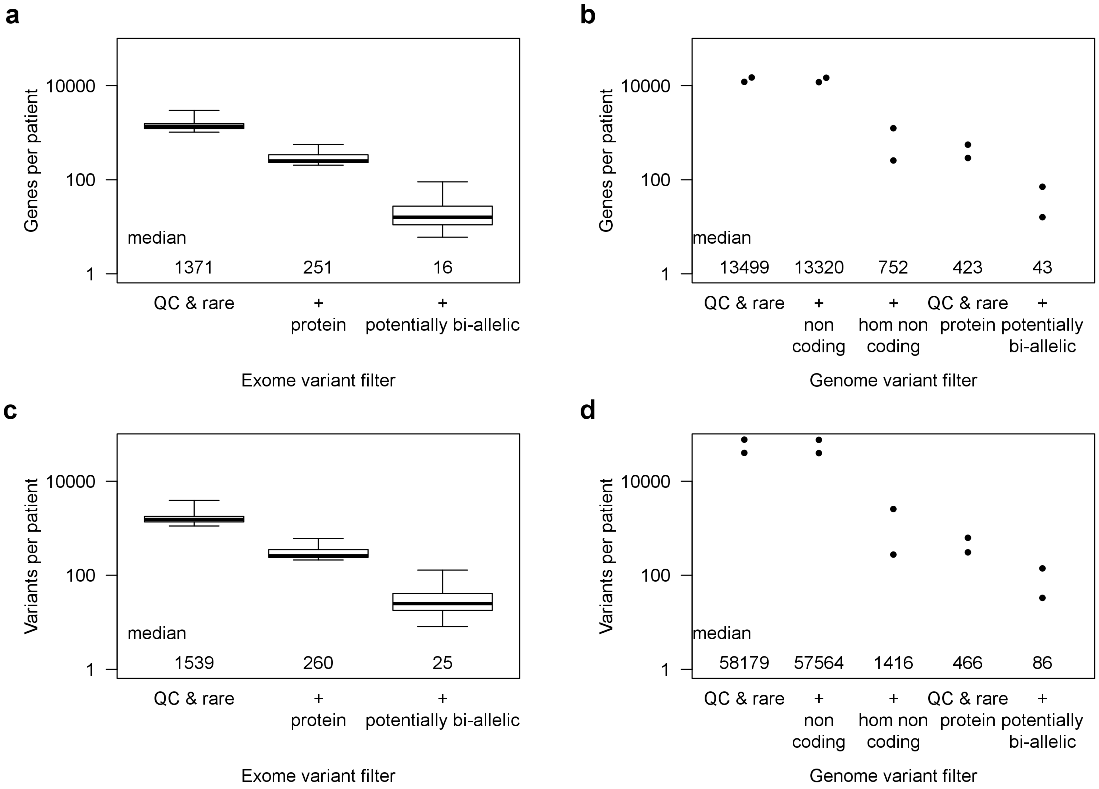

Variant filter for exome and genome sequencing. The different filtering steps are explained in detail in the Methods section. (a) Whole exome sequencing data filtered for candidate genes per patient that match the filter criteria. (b) Analog to (a), but for whole genome sequencing data. (c) Analog to (a), but for variants that match the filter criteria. (d) Analog to (b), but for variants that match the filter criteria. The whole genome sequencing data is based on the two *TIMMDC1* deficient patients #35791 and #66744.

10.Supplementary figure

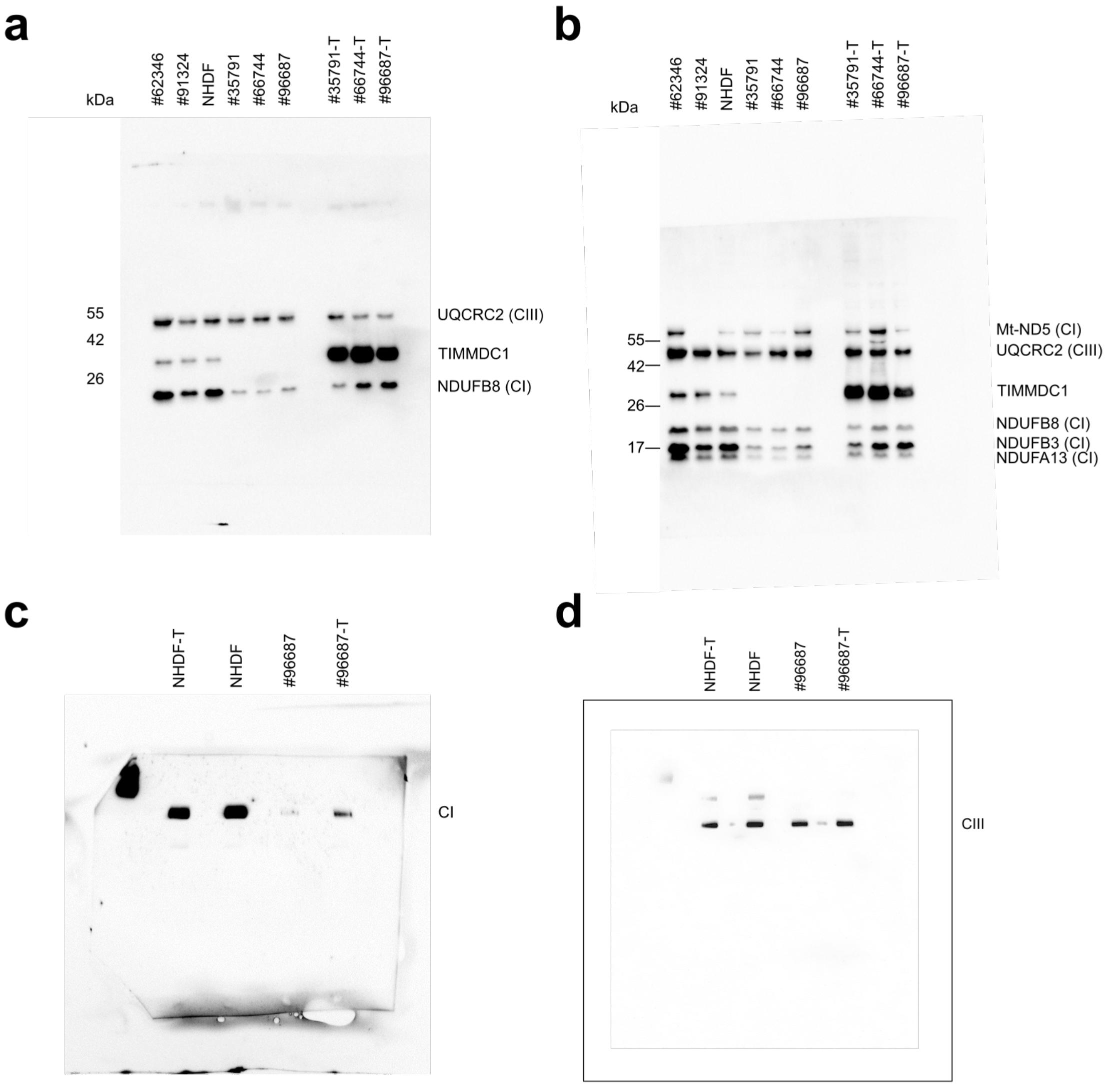

Uncropped blots for Fig. 2f and 2g. (a) upper panel of Fig. 2f. (b) lower panel of Fig. 2f. Western blot gels were loaded equally and run and blotted simultaneously. (c, d) Uncropped blue native PAGE blot of Figure 2g of the identical membrane stained (c) first with NDUFB8 and (d) subsequently with UQCRC2.

11.Supplementary figure

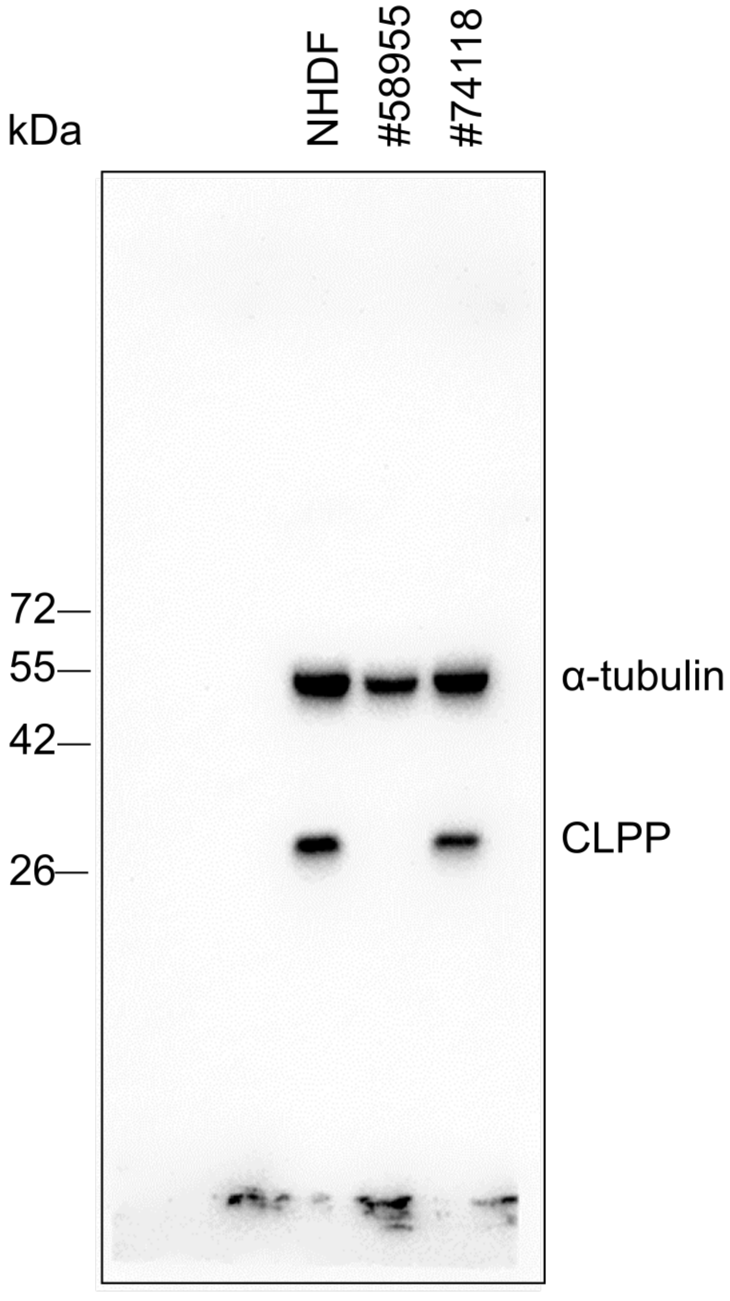

Uncropped Western blot for Supplementary Fig. 5.

Supplementary tables

1. *Supplementary Table: Sample numbers*

Method	Diagnosed	Not diagnosed	Total
RNA	57	48	105
RNA & WES	40	48	88
RNA & proteomics	11	20	31

Number of samples measured with the specified quantification method binned by their diagnosis status.

2. *Supplementary Table: Evaluation of RNA defects for diagnosed patients with WES*

Number of patients	Var gt	Var effect	Explained in paper by	Var type	Aber exp signi	Aber splice signi	Mono-allelic signi	comment
3	0/1; 0/1	missense; missense		snp; snp	-	-	-	
11	1/1	missense		snp	-	-	-	
3	0/1; 0/1	missense; stop	mae & exp	snp; snp	-	-	detected	
2	0/1; 0/1	missense; stop	mae & exp	snp; snp	-	-*	-	*one aber splice signi false positive
1	0/1; 0/1	missense; frame-shift	mae & frame	ins; snp	-	-	-	
1	0/1; 0/1	frame-shift;splice	splice & frame	ins; snp	-	-	detected*	*due to intron retention
7	1/1	splice	splice	snp	-	detected	-	
3	1/1	stop	exp	snp	detected	-	-	
1	1/1	stop	exp	snp	-	detected*	-	*observed exon skipping is unlikely to explain phenotype
1	0/1; 0/1	stop; exon deletion	exp	struc Var; snp	-	-	-	
2	1/1	frame-shift	exp	del	-	-	-	
2	0/1	frame-shift	exp	ins	-	-	-	
2	0/1; 0/1	frame-shift; del	exp	del; del	-	-	-	
1	1/1; 1/1	frame-shift; stop	exp	del; snp	-	-	-	

For each variant genotype (*Var gt*), effect (*Var effect*), and detection outcome (the 3 columns with the suffix "*_signi*" indicate which RNA defect could be detected for the causal gene) the *Number of patients* is given. Additionally, variant type is provided (*Var type*).

Supplementary Data

1. *Supplementary Data: Raw RNA gene counts*

Raw RNA gene counts per sample based on the UCSC gene annotation. The rows are labeled with the gene symbol and each column represents one sample (specified as *RNA_ID*). The counting process is described in detail in the methods part.

2. *Supplementary Data: Genes known to cause mitochondrial disorders*

This table reports names (*Gene*), Entrez gene IDs, the full gene name, the MIM number, and the link to the OMIM database of all genes known to cause mitochondrial disorders.

3. *Supplementary Data: Sample annotation*

This table provides an overview of all samples (*FIBROBLAST*_ID) included in this study. For solved cases the molecular diagnosis is reported in *DIAGNOSIS_GENE* with the predicted effect of the respective variant in *VARIANT_EFFECT. The column RNA_DEFECT_FOR_DIAGNOSIS_GENE describes, which of the three RNA strategies detected a defect in the DIAGNOSIS_GENE. HAS_EXOME* and *HAS_PROTEOME* are 'TRUE', if the data was available for analysis. For samples where WES was performed, the number of significant hits is given in *NUM_WES_SIGNI and the respective exome enrichment kit used for sequencing is given in EXOME_ENRICHMENT*. For each sample, the number of significant events observed for aberrant expression, aberrant splicing, and MAE is reported in *NUM_RNA_ABER_EXP_SIGNI, NUM_RNA_ABER_SPLICE_SIGNI,* and *NUM_MAE_SIGNI,* respectively.

4. *Supplementary Data: Raw protein LFQ values*

Raw LFQ intensities per sample and protein group. Each row represents a protein group, each column a sample by PROTEOME_ID. The file is extracted from a "proteinGroups.txt" output from the Max Quant Suite (Methods).

5. Supplementary Data: MaxLFQ parameter file

Parameters used for the quantification of peptide intensities with MaxLFQ. Each row is a pair of the name of the option and its value.

6. Supplementary Data: Aberrant splicing ranking for undiagnosed patients

Significant aberrant splicing events reported by our LeafCutter modification (Methods) for each sample (*FIBROBLAST*_ID) all splice clusters are given (SPLICE_CLUSTERID). The cluster ID is the unique identifier for each splicing event (multiple events can happen in one gene, *HGNCID*, in the same sample). Log-likelihood, nominal *P*-value and adjusted *P*-value are reported per cluster (SPLICE_LOGLR, SPLICE_PVALUE, SPLICE_PADJ, respectively). Gene information is reported analog to Supplementary Data 6 together with *the OTHER_DISEASE_GENES* column indicating if the gene is known in OMIM for a disease other than mitochondrial disorders. The table is sorted by adjusted *P*-value.

7. Supplementary Data: Candidates for undiagnosed patients

This table reports for each sample (*FIBROBLAST*_ID) that was not diagnosed previous to our study all genes (*HGNCID*) that have been discovered by either the WES filter (*WES_SIGNI*) or that showed a RNA defect (*RNA_ABER_EXP_SIGNI, RNA_ABER_SPLICE_SIGNI, MAE_IS_SIGNI*). Furthermore, it is annotated if the gene is known to cause mitochondrial disorders (*GENE_MITO_DISEASE*) and if its protein locates to mitochondrion (*GENE_GO_MITOCHONDRION*).

8. Supplementary Data: RNA normalization annotation

For each sample and RNA library (*FIBROBLAST*_ID, RNA_ID) *SEX*, *RNA_HOX_GROUP,* and *RNA_BATCH_GROUP* are reported.

9. Supplementary Data: Raw RNA split read counts

Raw RNA split read counts per sample for all exon junctions as part of a cluster defined by Leafcutter. The rows are labeled by the position of the junction and the cluster ID (chr:start:end:clusterID) assigned by Leafcutter. Each column represents one sample (specified as RNA ID).

Supplementary Note

1. Case reports

#35791 (TIMMDC1, c.[596+2146A>G]; [596+2146A>G], variants not listed in ExAC)

This boy was born at term to non-consanguineous Greek parents after uneventful twin pregnancy (dizygotic twins) via cesarean delivery (weight 2450 g, length 48 cm, head circumference 33 cm). He did not show obvious dysmorphia. Shortly after birth, he was noted to have muscular hypotonia and poor feeding behavior. During his first year of life developmental delay and failure to thrive became evident. Diagnostic work-up revealed sensorineural deafness and brain MRI showed enlarged ventricles and megacisterna magna. MR-spectroscopy as well as metabolic work-up in body fluids failed to detect specific abnormalities. Biochemical analysis of fibroblast and muscle tissue demonstrated a severe isolated complex I deficiency (16% of lowest control). He developed muscle wasting and a dyskinetic movement disorder, never achieving his developmental milestones and suffered from recurrent respiratory infections, finally leading to his death at the age of 30 months.

#66744 (TIMMDC1, c.[596+2146A>G]; [596+2146A>G], variants not listed in ExAC)

This boy was the only child of consanguineous parents from Northern Africa. Pregnancy, delivery and birth parameters were normal. Symptoms were first noted at the age of 6 months when he presented with muscular hypotonia, delayed acquisition of motor milestones, and nystagmus with altered electroretinogram and evoked visual potentials. An acute episode with abnormal eye movements, myoclonus, and loss of consciousness, followed by cerebellar syndrome, led to the diagnosis of Leigh syndrome, confirmed on a CT scan showing hypersignal in basal ganglia and mildly elevated lactate levels in blood. Subsequent NMR brain imaging was however normal. Biochemical analysis of muscle tissue showed a predominant complex I defect (15% of lowest control). Large-scale deletions and depletion of muscle mitochondrial DNA and common mtDNA mutations, including those involving *MT-ATP6*, were excluded. At one year of age, he presented with profound hypotonia, cerebellar syndrome with severe dysmetria, delayed mental development, and peripheral neuropathy. His lactate levels in blood and urine were repeatedly normal despite severe clinical condition. He died at 20 months of age.

#96687 (TIMMDC1, c.[596+2146A>G]; [596+2146A>G], variants not listed in ExAC)

This boy was born after uneventful pregnancy via spontaneous delivery to healthy, non-consanguineous parents from Germany (weight 4180 g, length 57 cm, head circumference 36 cm). Starting from the age of 3 months poor feeding behaviour, muscular hypotonia, and failure to thrive were noted. In the following, developmental delay and muscle wasting became evident. He showed severe cognitive/language impairment and never achieved ambulation. Starting from the age of four years, the patient developed severe therapy-resistant epilepsy. Brain MRI studies as well as metabolic work-up did not reveal any specific abnormalities. Of note, two older siblings died due to unexplained neurodegenerative disorders with severe epilepsy.

#73804 (MGST1)

This boy was born after uneventful pregnancy via spontaneous delivery to healthy, non-consanguineous German parents (weight 4050 g, length 56 cm, head circumference 37 cm). Soon after birth, his parents noted that he was less active and unusually quiet and did not fix or follow objects. Eye examinations suggested cortical or central blindness. In addition, hearing testing was repeatedly abnormal. During the first year of life severe developmental delay became evident and he developed epilepsy. Brain MRIs demonstrated rapidly progressive brain atrophy. Biochemical analysis of muscle tissue revealed a combined deficiency of complex III (75% of lowest control) and complex V (67% of lowest control). Metabolic work-up was otherwise normal. He never showed developmental progress and at the current age of 17 years he has severe intellectual disability and is wheelchair-bound. Of note, his clinical course as well as biochemical and neuroimaging findings, showed similarities to a patient suffering from thioredoxin 2 deficiency, a recently described disorder of mitochondrial oxidative stress regulation[1].

#58955 (CLPP, c.[661G>A];[661G>A], allele frequency $1.2*10^{-5}$ in ExAC)

This girl was the third child of healthy consanguineous parents (first cousins) of Turkish origin. She was born at term by spontaneous delivery after an uneventful pregnancy (weight 2755 g, length 49 cm, head circumference 37 cm). One brother, aged 17 years, is healthy; one brother, aged 13 years, suffers from sensorineural deafness diagnosed at age 2 years.

Her parents noted muscular weakness from the first week of life. When she presented at age 2 months, she was microcephalic (1 cm < 3rd percentile) and showed generalized muscular hypotonia. Echocardiography demonstrated mild hypertrophic cardiomyopathy. Metabolic analysis disclosed repeatedly metabolic acidosis with elevated lactate levels (2.8 to 9.0 mmol/l, normal < 2.3 mmol/l). Fumaric acid, 2-oxo-glutaric acid, and methylmalonic acid were mildly elevated in urine. Brain MRI at the age of 3 months showed no significant abnormality. Biochemical analysis of fresh muscle tissue showed decreased complex IV activity (26% of lowest control) and the coenzyme Q_{10} content was decreased (30% of lowest control). Muscle immunohistochemistry revealed near absence of immunoreactivity for complex IV subunits. Subsequently, progressive

developmental delay, persistent microcephaly, and deafness became evident. A percutaneous gastroenterostomy (PEG) was inserted for chronic feeding difficulties. She developed epilepsy (West-syndrome) and was treated with Topiramat, Levetiracetam, and Lamotrigen. Left ventricular hypertrophy initially progressed but stabilized under treatment with propranolol. She is currently 5 years of age and has microcephaly, deafness, severe psychomotor retardation, and moderate left ventricular hypertrophy.

#80256 (ALDH18A1, c.[1864C>T];[1988C>A], allele frequency $0.82*10^{-5}$;not listed in ExAC)

This boy was the first child of healthy non-consanguineous German parents. Prenatal ultrasound revealed intrauterine growth retardation. He was born without complications after 39 weeks of gestation (weight 2640 g, length 49 cm, head circumference 33.5 cm). Following birth, bilateral congenital cataract and multiple small haemangiomas were noted. In the first months of life, he developed muscular hypotonia, developmental delay, severe failure to thrive, and microcephaly. Brain MRI revealed hypoplasia of the corpus callosum, lack of insular opercularization, and reduced myelination. Moreover, the child developed drug-resistant epilepsy. A metabolic work-up showed no significant abnormalities. Muscle biopsy at age 2-½ years showed no significant morphological abnormality, whilst substrate oxidation and enzyme activities of mitochondrial complexes I – V were within normal limits. His onward clinical course was characterized by severe, global developmental delay, dyskinetic movement disorder, and epilepsy. He died at the age of 4 years from pneumonia and respiratory failure.

#62346 (MCOLN1, c.[681-19A>C];[832C>T], allele frequency $0.83*10^{-5}$; not listed in ExAC)

This boy was the third child of healthy non-consanguineous French parents. Pregnancy and delivery were uneventful whilst birth parameters and early psychomotor development of the child were normal. However, speech development was delayed, the patient acquiring language at the age of 4 years. At the age of 11 years, he began to

experience psychomotor regression and progressive visual loss due to degenerative retinopathy. He developed cerebellar ataxia, hyperreflexia, external ophthalmoparesis, bilateral corneal clouding, and abnormal behavior. The association of corneal clouding with a degenerative retinopathy and psychomotor regression was suggestive of mucolipidosis, but none of the enzymatic tests available for mucolipidosis type 1, 2, and 3 revealed an enzyme deficiency in blood leukocytes. Muscle biopsy showed moderate subsarcolemmal accumulation of mitochondria. At the current age of 47 years he has severe walking difficulties due to ataxia and blindness. On examination, he has cerebellar ataxia, hyperreflexia, external ophthalmoparesis predominating in vertical gaze, bilateral corneal clouding, and abnormal behavior (easily frightened, sometimes aggressive). Spontaneous speech is markedly reduced.

Informed consent was obtained from all affected individuals or their guardians in case of minor study participants. The study was approved by the ethical committee of the Technische Universität München.

2. R session Info

```
R version 3.2.2 (2015-08-14)
Platform: x86_64-pc-linux-gnu (64-bit)
Running under: Scientific Linux release 6.8 (Carbon)

locale:
 [1] LC_CTYPE=en_US.UTF-8          LC_NUMERIC=C
 [3] LC_TIME=en_US.UTF-8           LC_COLLATE=en_US.UTF-8
 [5] LC_MONETARY=en_US.UTF-8       LC_MESSAGES=en_US.UTF-8
 [7] LC_PAPER=en_US.UTF-8          LC_NAME=en_US.UTF-8
 [9] LC_ADDRESS=en_US.UTF-8        LC_TELEPHONE=en_US.UTF-8
[11] LC_MEASUREMENT=en_US.UTF-8    LC_IDENTIFICATION=en_US.UTF-8

attached base packages:
[1] stats4    parallel  stats     graphics  grDevices utils
datasets
[8] methods   base
```

```
other attached packages:
 [1] org.Hs.eg.db_3.2.3         RSQLite_1.0.0
 [3] DBI_0.3.1                  XLConnect_0.2-11
 [5] XLConnectJars_0.2-9        RCurl_1.95-4.8
 [7] bitops_1.0-6               VariantAnnotation_1.16.4
 [9] stringr_1.0.0              R.utils_2.2.0
[11] R.oo_1.20.0                R.methodsS3_1.7.1
[13] rtracklayer_1.30.2         RColorBrewer_1.1-2
[15] mgsa_1.18.0                rmarkdown_0.9.5
[17] LSD_3.0                    limma_3.26.8
[19] knitr_1.12.3               Hmisc_3.17-2
[21] ggplot2_2.1.0              Formula_1.2-1
[23] survival_2.38-3            gplots_2.17.0
[25] GenomicFeatures_1.22.13    GenomicAlignments_1.6.3
[27] Rsamtools_1.22.0           Biostrings_2.38.4
[29] XVector_0.10.0             geneplotter_1.48.0
[31] annotate_1.48.0            XML_3.98-1.4
[33] AnnotationDbi_1.32.3       lattice_0.20-33
[35] epitools_0.5-7             DT_0.1
[37] doMC_1.3.4                 iterators_1.0.8
[39] foreach_1.4.3              DEXSeq_1.16.10
[41] DESeq2_1.10.1              RcppArmadillo_0.6.500.4.0
[43] Rcpp_0.12.3                SummarizedExperiment_1.0.2
[45] Biobase_2.30.0             GenomicRanges_1.22.4
[47] GenomeInfoDb_1.6.3         IRanges_2.4.8
[49] S4Vectors_0.8.11           data.table_1.9.6
[51] biomaRt_2.26.1             BatchJobs_1.6
[53] BBmisc_1.9                 BiocParallel_1.4.3
[55] beeswarm_0.2.1             AnnotationHub_2.2.3
[57] BiocGenerics_0.16.1        rj_2.0.4-2

loaded via a namespace (and not attached):
 [1] httr_1.1.0                   tools_3.2.2
 [3] backports_1.0.0              R6_2.1.2
 [5] rpart_4.1-10                 KernSmooth_2.23-15
 [7] colorspace_1.2-6             nnet_7.3-12
 [9] gridExtra_2.2.1              chron_2.3-47
```

```
[11] rj.gd_2.0.0-1                sendmailR_1.2-1
[13] formatR_1.3                  caTools_1.17.1
[15] scales_0.4.0                 checkmate_1.7.2
[17] genefilter_1.52.1            digest_0.6.9
[19] foreign_0.8-66               base64enc_0.1-3
[21] htmltools_0.3                BSgenome_1.38.0
[23] htmlwidgets_0.6              BiocInstaller_1.20.3
[25] shiny_0.13.1                 jsonlite_0.9.19
[27] hwriter_1.3.2                gtools_3.5.0
[29] acepack_1.3-3.3              magrittr_1.5
[31] futile.logger_1.4.1          munsell_0.4.3
[33] viridis_0.3.3                yaml_2.1.13
[35] stringi_1.0-1                zlibbioc_1.16.0
[37] fail_1.3                     plyr_1.8.3
[39] grid_3.2.2                   gdata_2.17.0
[41] splines_3.2.2                locfit_1.5-9.1
[43] codetools_0.2-14             futile.options_1.0.0
[45] evaluate_0.8.3               latticeExtra_0.6-28
[47] lambda.r_1.1.7               httpuv_1.3.3
[49] gtable_0.2.0                 mime_0.4
[51] xtable_1.8-2                 rJava_0.9-8
[53] cluster_2.0.3                statmod_1.4.24
[55] brew_1.0-6                   interactiveDisplayBase_1.8.0
```

Supplementary Methods

1. RNA sequencing count normalization

Hierarchical clustering revealed three main clusters that could not be linked to biological or technical properties of the samples. These clusters were considered as groups of unknown technical variation. Furthermore, 5 HOX genes were among the 150 genes with most variable expression (3.3% w.r.t. 0.2% HOX genes in all 12,680 genes). HOX genes are important regulators of the body plan during development of the anterior-

posterior axis [2]. Since the fibroblast cell lines are taken from different body parts depending on the clinic, the first diagnosis, and other factors, we hypothesized that the expression pattern of the HOX genes is a good proxy for the body parts of the biopsy. To control for this effect we performed hierarchical clustering of the RNA expression across all samples based only on the HOX genes (identified as genes with names starting with "HOX") which revealed four major sample clusters (Supplementary Fig. 1).

2. *Mass spectrometric sample preparation*

We performed quantitative proteomics from a second aliquot of cells taken at the same time as the RNA-seq aliquot. Mass spectrometric sample preparation was done similar to an earlier study[3]. Briefly, cells were lysed in SDC lysis buffer (1% sodium deoxycholate, 10 mM TCEP, 40 mM CAA, 100 mM Tris pH 8.5), boiled for 10 min at 95ºC, sonicated and diluted 1:1 with water for LysC and trypsin digestion. The dilution buffer contained appropriate amounts of proteolytic enzyme to ensure a ratio of 1:50 (µg enzyme / µg protein). Digestion was performed at 37°C overnight. Peptides were acidified, loaded on SDB-RPS (poly-styrenedivinylbenzene) material and eluted. Eluted peptides were collected in autosampler vials and dried using a SpeedVac centrifuge (Eppendorf, Concentrator plus, 5305 000.304). Peptides were resuspended in buffer A* (2% ACN, 0.1% TFA) and were sonicated (Branson Ultrasonics, Ultrasonic Cleaner Model 2510).

3. *Mass spectrometric data acquisition*

2 µg of peptides per sample were loaded for 100 min gradients separated on a 50 cm column with 75 µm inner diameter in-house packed with 1.9 µm C18 beads (Dr. Maisch GmbH). Reverse phase chromatography was performed at 50ºC with an EASY-nLC 1000 ultra-high pressure system (Thermo Fisher Scientific) coupled to the Q Exactive HF[4] mass spectrometer (Thermo Fisher Scientific) via a nanoelectrospray source (Thermo Fisher Scientific). Peptides were loaded in buffer A (0.1% volume/volume formic acid) and eluted with a nonlinear gradient. Operational parameters were real-time monitored by the SprayQC software[5]. Raw files were analysed by the software MaxQuant[6] (version

1.5.3.2) and peak lists were searched against the Homo sapiens Uniprot FASTA database (Version 2014/4) and a common contaminants database (247 entries) by the Andromeda search engine[7]. Label-free quantification was done using the MaxLFQ algorithm[8] (for detailed parameters see Supplementary Data 9) integrated into MaxQuant.

4. *Processing of proteome intensities*

The LFQ intensities and gene names were extracted for 6,566 protein groups from the MaxQuant output file *proteinGroups.txt*. For protein groups with more than one member, the first member was chosen to represent the group as single protein with a distinct gene name (similar to earlier studies[9]). MaxLFQ intensities of 0 actually represent non-quantified peaks and were therefore replaced with missing values (NA). The 10 samples that had a frequency of missing values higher than 50% were considered bad quality and were discarded. Furthermore, proteins were discarded because they had no gene name assigned (n=198), were not the most abundant among their duplicates (n=295), were not expressed in any sample (n=93), because their 95th percentile was not detected (n=549), which was also considered as not expressed, analogously to RNA filtering. Finally, 5,431 proteins and 31 samples were considered for further analysis (Supplementary Data 4).

5. *Computing protein fold changes and differential expression*

Since the mass spectrometric measurements of all samples were done in a single run, no technical artifacts could be found with a hierarchical clustering. Protein differential expression for each patient compared to the others was tested using moderated T-test approach as implemented in the R/Bioconductor limma package[10]. The transcriptome covariates for sex and HOX effects were used in the linear model for normalization.

6. *Sanger sequencing*

DNA sequences were amplified by PCR using the Qiagen Taq DNA Polymerase Kit (Qiagen, Hilden, Germany) and a PeqStar thermal cycler (PeqLab Biotechnology, Erlangen, Germany) using the following primers:

- *TIMMDC1*_Intron5_forward, 5’- GGGCATAATATATTCACAGTTGAGG-3’;
- *TIMMDC1*_Intron5_reverse, 5’- ACAACAAAAGCAATGGCAGCA-3’;

- *ALDH18A1*_Exon14-15_forward,
  5'- TGTAAAACGACGGCCAGTTCTGTAAAAGGGAAGCTGCTG-3';
- *ALDH18A1*_Exon14-15_reverse,
  5'- CAGGAAACAGCTATGACCGCTGTGCCTGGTCTAATTCC-3';
- *ALDH18A1*_Exon16_forward,
  5'- TGTAAAACGACGGCCAGTTTGGGCTGTGGTTTTACAGG-3';
- *ALDH18A1*_Exon16_reverse,
  5'- CAGGAAACAGCTATGACCGCAGGATCAGAAAGCAGC-3'.

PCR was purified using the NucleoFast® 96 PCR Plate (MACHEREY-NAGEL, Düren, Germany). The purified PCR product was used for subsequent cycle sequencing using the ABI BigDye Terminator v.3.1 Cycle Sequencing kit (Life Technologies, Carlsbad, CA, USA). For purification, the sequencing reaction was precipitated with 25 µL 100% ethanol for 15 min in the dark followed by centrifugation at 3000 g for 30 min at RT. The pellet was washed with 125 µL 70% ethanol, centrifuged at 2000 g for 10 min, and left to dry at RT in the dark. Subsequently, the pellet was resuspended in 25 µL ultrapure H2O, transferred to a microtiter plate, and placed into the automated ABI 3730 sequencer. Resulting sequences were analyzed using Staden Package (http://staden.sourceforge.net).

7. *Whole genome sequencing and variant prioritization*

Whole genome sequencing libraries were prepared using Illumina's TruSeq DNA PCR-Free Library Prep Kit and sequenced as 150 bp paired-end runs on an Illumina HiSeq4000 platform. Resulting reads were aligned using BWA-MEM v0.7.5a-r405[11]. Variant calling was performed with GATK's HaplotypeCaller and the genotypes were then jointly called with GATK's GenotypeGVCF (GATK version: v3.5-0-g36282e4)[12]. The same variant annotation and quality filtering steps as in the whole exome sequencing pipeline were applied (see Methods). Intergenic variants were discarded which are defined as being more than 5kb away from any gene. In addition to the ExAC database filter, a variant is defined as rare with a minor allele frequency < 0.001 within the 1000 Genome Project[13]. Besides the already defined filtering criteria *rare*, *protein affecting*,

and *potentially biallelic* we filtered for non-coding variants, which are annotated as *intronic*, *5' UTR*, or *3' UTR* by VEP from Ensembl, and for homozygous non-coding variants.

8. Metabolomics

Blood levels of metabolites of the proline pathway and urea cycle were determined as part of a non-targeted metabolomics experiment on 143 patients (including #80256) with mitochondrial diseases and 97 healthy controls. We used a metabolomics platform that has been established by Metabolon Inc. and is based on mass spectrometry coupled to liquid (LC-MS/MS). Sample preparation, analytical protocols, identification of metabolites, and processing of the raw ion counts have been established previously[14–16], but are also described in the following:

Briefly, plasma samples, which were stored at -80°C prior to analysis, were thawed on ice. For metabolite extraction and protein precipitation, 475 µL methanol, containing four recovery standards to monitor the extraction efficiency, were added to 100 µL of the plasma. After centrifugation, the supernatant was split into 4 aliquots of 100 µL each onto two 96-well microplates. The first 2 aliquots were used for LC-MS/MS analysis in positive and negative electrospray ionization mode. Two further aliquots were kept as a reserve. The extracts were dried on a TurboVap 96 (Zymark, Sotax, Lörrach, Germany). Prior to LC-MS/MS in positive ion mode, the samples were reconstituted with 50 µl 0.1% formic acid, whereas samples analyzed in negative ion mode were reconstituted with 50 µl 6.5 mM ammonium bicarbonate, pH 8.0. Reconstitution solvents for both ionization modes contained internal standards that allowed monitoring of instrument performance and also served as retention reference markers. To minimize human error, liquid handling was performed on a Hamilton Microlab STAR robot (Hamilton Bonaduz AG, Bonaduz, Switzerland).

LC-MS/MS analysis was performed on a linear ion trap LTQ XL mass spectrometer (Thermo Fisher Scientific GmbH, Dreieich, Germany) coupled with a Waters Acquity UPLC system (Waters GmbH, Eschborn, Germany). Two separate columns (2.1 x 100 mm Waters BEH C18, 1.7 µm particle-size) were used either for acidic (solvent A: 0.1% formic acid in water, solvent B: 0.1% formic acid in methanol) or for basic (A: 6.5 mM

ammonium bicarbonate, pH 8.0, B: 6.5 mM ammonium bicarbonate in 95% methanol) mobile phase conditions, optimized for positive and negative electrospray ionization, respectively. After injection of the sample extracts, the columns were developed in a gradient of 99.5% A to 98% B over an 11 min run time at 350 µL/min flow rate. The eluent flow was directly run through the ESI source of the LTQ XL mass spectrometer. The mass spectrometer analysis alternated between MS and data-dependent MS/MS scans using dynamic exclusion and the scan range was from 80-1000 m/z.

Metabolites were identified by Metabolon, Inc. from the LC-MS/MS data by automated multiparametric comparison with a proprietary library containing retention times, m/z ratios, and related adduct fragment spectra.

To account for instrument inter-day differences, the raw ion counts detected for each metabolite were divided by their median per run-day. Furthermore, a log transformation of base 10 was applied as measured metabolite levels mostly follow a log-normal distribution.

9. Cellular ROS production

Intensity of hydroethidine (HEt) oxidation products as a measure of cellular ROS production was quantified in living skin fibroblasts using epifluorescence microscopy as described previously[17].

Supplementary References

1. Holzerova, E. *et al.* Human thioredoxin 2 deficiency impairs mitochondrial redox homeostasis and causes early-onset neurodegeneration. *Brain* **139,** 346–54 (2016).
2. Lewis, E. B. A gene complex controlling segmentation in Drosophila. *Nature* **276,** 565–70 (1978).
3. Kulak, N. A., Pichler, G., Paron, I., Nagaraj, N. & Mann, M. Minimal, encapsulated proteomic-sample processing applied to copy-number estimation in eukaryotic cells. *Nat. Methods* **11,** 319–324 (2014).
4. Scheltema, R. A. *et al.* The Q Exactive HF, a Benchtop mass spectrometer with a

pre-filter, high-performance quadrupole and an ultra-high-field Orbitrap analyzer. *Mol. Cell. Proteomics* **13,** 3698–708 (2014).

5. Scheltema, R. A. & Mann, M. SprayQc: A Real-Time LC-MS/MS quality monitoring system to maximize uptime using off the shelf components. *J. Proteome Res.* **11,** 3458–3466 (2012).
6. Cox, J. & Mann, M. MaxQuant enables high peptide identification rates, individualized p.p.b.-range mass accuracies and proteome-wide protein quantification. *Nat. Biotechnol.* **26,** 1367–72 (2008).
7. Cox, J. *et al.* Andromeda: A peptide search engine integrated into the MaxQuant environment. *J. Proteome Res.* **10,** 1794–1805 (2011).
8. Cox, J., Hein, M. Y., Luber, C. a & Paron, I. Accurate proteome-wide label-free quantification by delayed normalization and maximal peptide ratio extraction, termed MaxLFQ. *Mol. Cell. ...* **13,** 2513–2526 (2014).
9. Cheng, Z. *et al.* Differential dynamics of the mammalian mRNA and protein expression response to misfolding stress. *Mol. Syst. Biol.* **12,** 855–855 (2016).
10. Ritchie, M. E. *et al.* Limma powers differential expression analyses for RNA-sequencing and microarray studies. *Nucleic Acids Res.* **43,** e47 (2015).
11. Li, H. Aligning sequence reads, clone sequences and assembly contigs with BWA-MEM. *arXiv* 1–3, http://arxiv.org/abs/1303.3997 (2013).
12. McKenna, A. *et al.* The Genome Analysis Toolkit: a MapReduce framework for analyzing next-generation DNA sequencing data. *Genome Res.* **20,** 1297–303 (2010).
13. 1000 Genomes Project Consortium *et al.* A global reference for human genetic variation. *Nature* **526,** 68–74 (2015).
14. Evans, A. M., DeHaven, C. D., Barrett, T., Mitchell, M. & Milgram, E. Integrated, nontargeted ultrahigh performance liquid chromatography/electrospray ionization tandem mass spectrometry platform for the identification and relative quantification of the small-molecule complement of biological systems. *Anal. Chem.* **81,** 6656–6667 (2009).
15. Dehaven, C. D., Evans, A. M., Dai, H. & Lawton, K. A. Organization of GC/MS and LC/MS metabolomics data into chemical libraries. *J. Cheminform.* **2,** (2010).

16. Shin, S.-Y. *et al.* An atlas of genetic influences on human blood metabolites. *Nat. Genet.* **46,** 543–550 (2014).

17. Forkink, M., Smeitink, J. a M., Brock, R., Willems, P. H. G. M. & Koopman, W. J. H. Detection and manipulation of mitochondrial reactive oxygen species in mammalian cells. *Biochim. Biophys. Acta* **1797,** 1034–44 (2010).
